# Characteristics of the BDS Carrier Phase Multipath and Its Mitigation Methods in Relative Positioning

**DOI:** 10.3390/s17040796

**Published:** 2017-04-07

**Authors:** Wujiao Dai, Qiang Shi, Changsheng Cai

**Affiliations:** Department of Surveying and Geo-informatics, Central South University, Changsha 410083, China; promise_shi@163.com (Q.S.); csucai@csu.edu.cn (C.C.)

**Keywords:** BDS, carrier phase, multipath effects, double difference, static baseline solution

## Abstract

The carrier phase multipath effect is one of the most significant error sources in the precise positioning of BeiDou Navigation Satellite System (BDS). We analyzed the characteristics of BDS multipath, and found the multipath errors of geostationary earth orbit (GEO) satellite signals are systematic, whereas those of inclined geosynchronous orbit (IGSO) or medium earth orbit (MEO) satellites are both systematic and random. The modified multipath mitigation methods, including sidereal filtering algorithm and multipath hemispherical map (MHM) model, were used to improve BDS dynamic deformation monitoring. The results indicate that the sidereal filtering methods can reduce the root mean square (RMS) of positioning errors in the east, north and vertical coordinate directions by 15%, 37%, 25% and 18%, 51%, 27% in the coordinate and observation domains, respectively. By contrast, the MHM method can reduce the RMS by 22%, 52% and 27% on average. In addition, the BDS multipath errors in static baseline solutions are a few centimeters in multipath-rich environments, which is different from that of Global Positioning System (GPS) multipath. Therefore, we add a parameter representing the GEO multipath error in observation equation to the adjustment model to improve the precision of BDS static baseline solutions. And the results show that the modified model can achieve an average precision improvement of 82%, 54% and 68% in the east, north and up coordinate directions, respectively.

## 1. Introduction

Currently, BeiDou Navigation Satellite System (BDS) is providing position, navigation and timing services for most of the Asia-Pacific area. It consists of geostationary earth orbit (GEO) satellites, inclined geosynchronous orbit (IGSO) satellites and medium earth orbit (MEO) satellites. Current studies have confirmed that the position precision of BDS is as good as that of Global Positioning System (GPS) [1,2]. Similar to GPS, distance-dependent errors, such as tropospheric delays, ionospheric delays and orbital errors, can be mostly removed by the double-difference operation in short-baseline (e.g., <5 km) positioning. However, site-dependent multipath errors cannot be eliminated or reduced by this technique. Thus, the multipath errors become a major error source for BDS relative positioning.

GPS technology has developed for many years, and various techniques have been studied to mitigate its multipath, which can be classified to three groups [3]. The first one is using multipath-rejecting GPS antennas, like the chokering antenna and the advanced pinwheel compact controlled reception pattern antenna [4]. Those antennas can effectively weaken the multipath effects, but they are large and expensive. What’s more, they are ineffective in dealing with signals with high elevation angles. The second one is the receiver-internal correlation techniques, for example, narrow correlator [5], Multipath Mitigation Technology [6], and Multipath Mitigation Delay Lock Loop [7]. These techniques can effectively reduce the code and carrier phase multipath effects, but meanwhile incur high computational costs and require high-level hardware. The third one is post-processing algorithms including weighting observations based on signal-to-noise ratio (SNR) or carrier-to-noise power-density (C/N0) [8] and filter-based approaches, such as the Wavelet Filter [9], Adaptive Filter [10], Vondrak filter [11], and sidereal filter [12]. These filters can effectively mitigate the multipath errors, among which, the sidereal filtering is most widely used, since it is immune to the effect of frequency aliasing [3]. Due to similar satellite geometry, multipath has an intrinsic repeatability in the cycle of a sidereal day when the surroundings of the GPS antenna remain unchanged. Therefore, sidereal filtering is feasible to mitigate multipath errors in both the coordinate and the observation domain [13].

For sidereal filtering in the coordinate domain, multipath is mitigated using the repeatability of multipath sequences on a daily basis at a fixed station. The orbital periods of satellites have been discussed in depth in some literatures. Seeber et al. [14] was the first to notice that the repeat cycle of the satellite is not a sidereal day, and it varies with satellites. Axelrad et al. [15] and Ragheb et al. [16] showed that the actual satellite geometry repeat interval is slightly shorter than generally assumed. Larson et al. [17] showed that the repeat time is averagely 246s shorter than a solar day and proposed the aspect repeat time adjustment (ARTA) method to estimate the time shifts using GPS coordinate time series. Once the orbit repeat time is determined accurately, a filter value is deducted from the baseline solution at each epoch and a time shift is made to calibrate multipath errors for subsequent measurements [16,18].

When the sidereal filtering is carried out in the observation domain, the multipath errors can be extracted from single differenced or double differenced residuals. Alber et al. [19] obtained single path phase delays from GPS double differences by using a transfer matrix. Ragheb et al. [16] used the autocorrelation of double differenced phase residuals to determine the optimal lag and then subtracted multipath errors derived from double differenced residuals from the observations on subsequent days. Zhong et al. [12] used a new sidereal filtering method based on GPS single differences to mitigate GPS multipath, which considered the different orbital repeat periods of GPS satellites. Recently, Dong [20] proposed a multipath hemispherical map (MHM) model based on the multipath spatiotemporal repeatability. This method is efficient and suitable for real-time processing, avoiding frequent calculation of the accurate repeat period of multipath signals for each satellite.

As discussed above, most relating researches are based on GPS observations [12,16,18] and only a few preliminary investigations have conducted on BDS multipath [21,22,23,24,25,26,27]. Wu et al. [21] and Shi et al. [22] investigated the characteristics of BDS pseudo-range multipath, but failed to consider the characteristics of BDS carrier phase. Yang et al. [24] proposed a method to mitigate code multipath by installing a wave-absorbing shield around the antennas of GNSS tracking sites, which did not have universal applicability. Ning et al. [25] investigated the characteristic of code bias of the BDS GEO satellites and improved the positioning accuracy of single point positioning (SPP) and precise point positioning (PPP). Xie et al. [26] mitigated multipath bias using a dual-polarization antenna by proposing DP-SIMLE algorithm, from the perspective of antenna design, which did not have universal applicability for surveyor. Jin et al. [27] used BDS multipath signals to estimate sea level changes instead of mitigating them. And their result shows a good agreement with Tide Gauge. Ye et al. [23] investigated the characteristics of BDS carrier phase multipath and mitigated the multipath errors by sidereal filtering based on single differences. This process needs recover the single differenced residuals from double differenced residuals using an independent constraint, based on the assumption that the sums of observed satellites' single differenced residuals are zero. However, the carrier phase multipath errors of GEO satellites are systematic, which is not suitable to recover the single differenced residuals from double differenced residuals using a simple "zero mean" assumption, as it will add new errors to BDS positioning results. Therefore, we use the double differences method to study the BDS multipath.

This study aims to investigate the characteristics of BDS multipath, analyze the multipath effects on BDS relative positioning and propose the BDS multipath mitigation methods to improve the precision of BDS positioning.

## 2. Basic Principle and Calculation Method of Multipath

As discussed in section “Introduction”, the characteristics of BDS are different from that of GPS. In order to study the characteristics of BDS in detail, the basic principle and the calculation method of BDS multipath are presented in this section.

### 2.1. Basic Principle of Multipath

BDS satellite signals coming from both the direct and reflect directions are referred to as multipath signals. In practice, objects near the receiver reflect direct signals, and these reflected signals go through a longer path than the original, causing large propagation delay [28].

**Figure 1:**
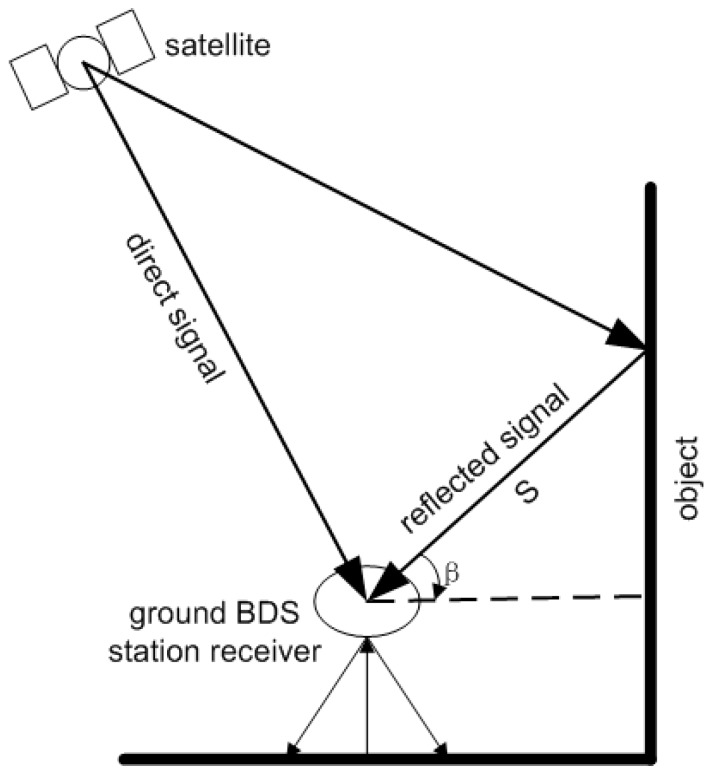
Principle of multipath.

Assuming there is only one multipath signal (Figure 1), the received signal is the superposition of the direct and the reflected signals, which can be expressed as follows [29]:

(1) $$S_{total}=A\sin(\omega_{0}t)+\alpha A\sin(\omega_{0}t+\Delta )=\alpha_{c}A\sin(\omega_{0}t+\Delta_{c})$$

where A is the signal amplitude, $\omega_{0}$ is the angular frequency, Δ is the phase shift between the direct and the reflected signals, $\alpha_{c}$ is the attenuation coefficient. These variables are written as follows:

(2) $$\left\{ \begin{array}{l} \alpha_{c}=\sqrt{1+2\alpha \cos\theta +\alpha^{2}}\\ \Delta_{c}=\arctan(\alpha \sin\Delta /(1+\alpha \cos\Delta ))\\ \Delta =4\pi \cdot s\cdot \sin\beta /\lambda \end{array}\right.$$

where s is the distance between the antenna and the reflector, β is the incident angle of the reflection signal, λ is the signal wavelength. When the reflector is known, the multipath error can be written as follows [30]:

(3) $$S_{\mathrm{multi}}=\frac{\Delta_{c}}{2\pi }\lambda =\frac{\lambda }{2\pi }\arctan(\frac{\alpha \sin\Delta }{1+\alpha \cos\Delta })$$

It can be seen from Equation (3) that the maximum multipath error is 0.25 λ, corresponding to 4.8 cm for BDS B1 carrier phase observation according to its wavelength.

### 2.2. Calculation of Carrier Phase Multipath

Since the double differenced (DD) technique can mitigate most ionospheric delays, tropospheric delays and orbit errors in short-baseline relative positioning, it has been widely used for baseline processing. The DD carrier phase model for a single satellite system can be expressed as:

(4) $$\lambda_{j}\nabla \Delta \Phi_{ij}^{p}=\nabla \Delta \rho_{ij}+\lambda_{j}\nabla \Delta N_{ij}+\nabla \Delta \delta_{\mathrm{multipath}}+\;\nabla \Delta \varepsilon_{c}$$

where $\nabla \Delta \Phi_{ij}^{p}$, $\nabla \Delta \rho_{ij}$, $\nabla \Delta N_{ij}$, $\nabla \Delta \delta_{\mathrm{multipath}}$ and $\nabla \Delta \varepsilon_{c}$ are the variables. ∇Δ is the DD operator; $\lambda_{j}$ is the wavelength; $\nabla \Delta \Phi_{ij}^{p}$ denotes the DD carrier phase observations; $\nabla \Delta \rho_{ij}$ is the DD distance between the satellites and receivers; $\nabla \Delta N_{ij}$ is the DD integer ambiguities; $\nabla \Delta \delta_{\mathrm{multipath}}$ is the multipath error, and $\nabla \Delta \varepsilon_{c}$ expresses the noise. In this study, elevation stochastic model is used in static baseline solution. It is expressed as $\sigma^{2}=a^{2}+b^{2}/{\left(\sin(Elev)\right)}^{2}$, where a and b are the model coefficients, and Elev is the elevation angle of satellite.

The DD carrier phase residuals are mainly the multipath errors. The DD carrier phase linear observation can be expressed as:

(5) V=Ax+By−L

where x and y are the variables. A and B are the design matrix; x is the baseline vector; y is the integer DD ambiguities vector; L is the constant term of carrier phase; V is the DD residuals including DD multipath errors and noise.

The DD observation residuals are obtained by the following steps: (1) First, the observations collected in a weak multipath environment were processed in a static mode to obtain the real-valued baseline vectors x. (2) The float values of integer ambiguities y were calculated by substituting the real-valued baseline vectors x into Equation (5) for the observations collected in multipath-rich environments. (3) Fix the integer ambiguities y using rounding method. (4) Finally, substitute x and y into Equation (5) at every epoch for the observations collected in multipath-rich environments to obtain the DD observation residuals.

## 3. Data Collection

**Figure 2:**
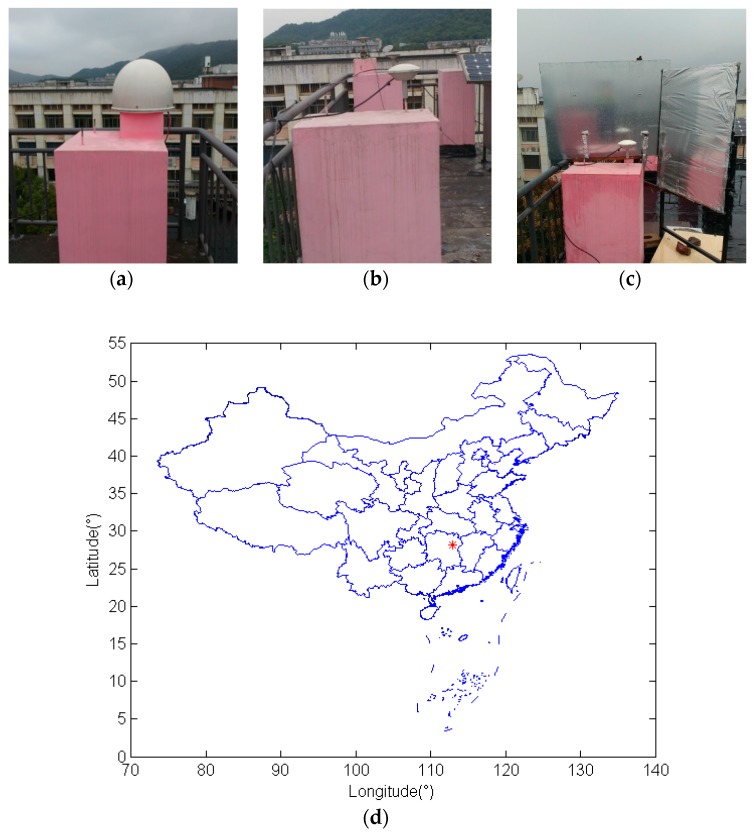
Photographs of the base station (**a**); the rover station in weak multipath environment (**b**); the rover station in multipath-rich environments (**c**); and the approximate location (28.170365° N, 112.925150° E, 69.705 m) of rover station on the map of China (**d**).

Datasets from 19 October 2015 to 19 Novermber 2015 were collected at a sampling rate of 1 Hz with an elevation mask angle of 0° on the rooftop of the Mining Building at the Central South University, China, using “Trimble Net R9”GNSS receivers with “Zephyr Geodetic2” antennas. To investigate the characteristics and effects of BDS carrier phase multipath on static baseline solutions, measurements were made simultaneously with two receivers, one base station (Figure 2a) and one rover station (Figure 2b). The baseline between these two stations is 6.5 m. Data were firstly collected in a weak multipath environment during the first seven days at the rover station, and then two reflectors were mounted on the north (Figure 2c) to create a stronger multipath environment, which is similar to Ye [23]. The red asterisk in Figure 2d represents the approximate location of rover station on the map of China.

The DD observation residuals were obtained using our in-house GNSSDMS software, which was designed for short-baseline deformation monitoring in both the static and dynamic modes. In this study, only the B1 carrier phase observations were used. In order to analyze the effects of multipath on BDS static baseline solutions, two baselines were processed in the static model using BDS and GPS observations, respectively, with an elevation mask angle of 10°. In addition, the diffracted observation data were eliminated in data preprocessing using a deleting satellite method. In this method, an obstacle model around the antenna should be established at first, which is based on the geometry between the reflectors and the rover antenna. Then the azimuths and elevations of the satellites are calculated. If one satellite is masked by the reflectors, but the observations of this satellite are recorded in rover observation file, then we can conclude these observations come from the diffracted signals of this satellite, and delete them directly in data preprocessing.

## 4. BDS Multipath Characteristics Analysis

**Figure 3:**
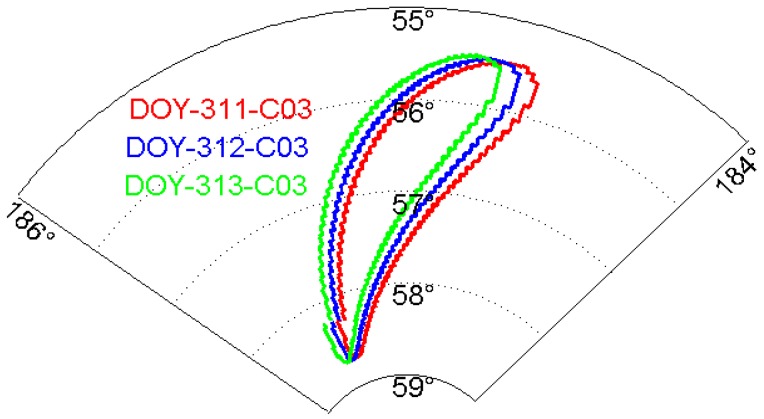
Trajectory of C03 for day of the year (DOY) 311-313 in the elevation and azimuth domain.

**Figure 4:**
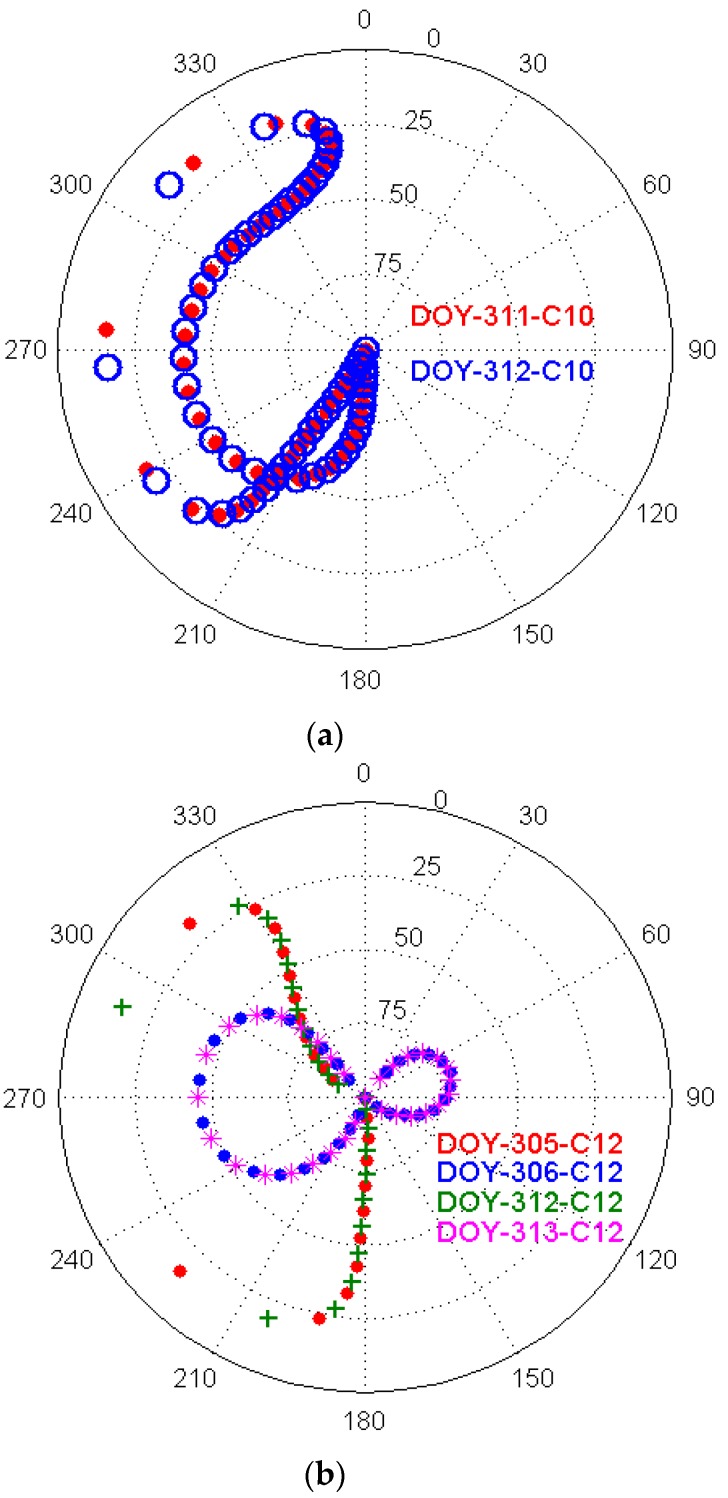
Trajectory of C10 (**a**) and for DOY 311-312 and C12 (**b**) for DOY 305-313 in the elevation and azimuth domain.

In order to investigate the repeatability of BDS multipath effects, we analyzed the repeatability of its three constellations GEO, IGSO and MEO. Their trajectories are shown in Figure 3 and Figure 4, which are plotted with respect to the varying satellite elevations and azimuths.

It can be seen from Figure 3 that GEO satellites are not stationary but oscillate in a small area. Figure 4a shows the elevation and azimuth changes of IGSO C10 over two consecutive days (311–312). Clearly, the movement range of IGSO satellites is larger than that of GEO satellites, and their trajectories almost overlap each other during the consecutive days. Figure 4b shows the elevation and azimuth changes of MEO C12 for DOY 305, 306, 312 and 313. Clearly, the trajectories of MEO satellites in DOY 305 and 312 are similar, while the one in DOY 306 resembles that in DOY 313. This suggests that the orbital repeat periods of MEO satellites are approximately seven sidereal days.

Two methods can be employed to estimate the daily shift of the BDS satellite geometry. One method uses the law of Keplerian orbit to compute the period from the semimajor axis which are given in the broadcast ephemeris, and the other method finds the actual repeat geometry for a selected location and identify the associated time shift [15]. As GEO satellites move repeatedly in a very small range, the dot products of two user-to-satellite line of sight vectors are almost similar all the time. So the second method is not applicable to GEO satellites. We selected the first method to calculate the time shift (Ta) of GEO, IGSO and MEO satellites, and the second method for the time shift (Tg) of IGSO and MEO satellites. The results are displayed in Table 1 and Table 2.

As the comparison between Ta and Tg in Table 1 and Table 2 shows, there is a great agreement between IGSO and MEO satellites. The daily shift of GEO and IGSO satellites geometry is similar to that of GPS which is approximately 244–246 s [15], but the one of MEO satellites geometry is different from GPS.

**Figure 5:**
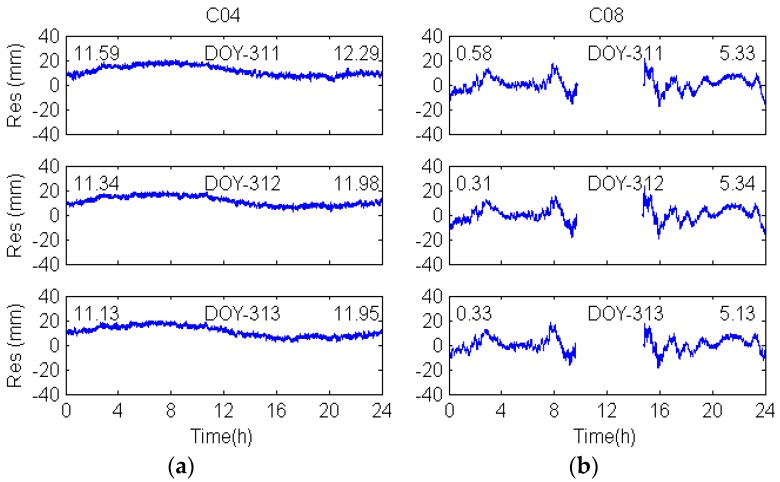
Denoised DD residuals of GEO (**a**) and IGSO (**b**) for three consecutive days. Numbers (unit: mm) in the top left and right corners represent the mean and RMS value of the DD residuals, respectively.

**Figure 6:**
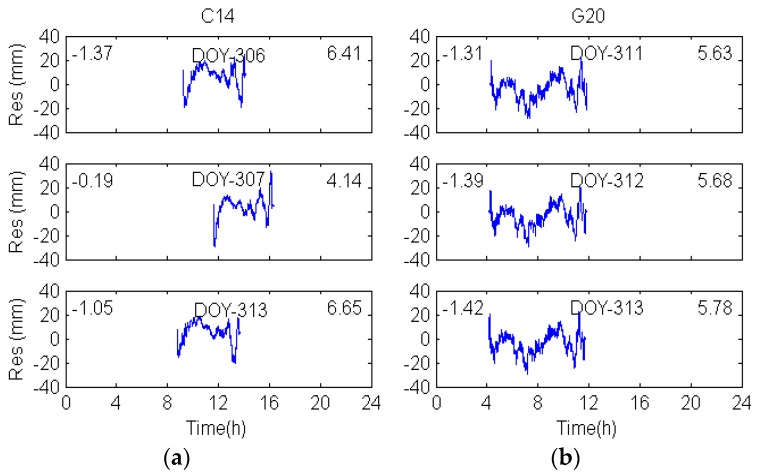
Denoised DD residuals for MEO-C14 (**a**) and GPS-G20 (**b**).

To compare the multipath effects of different constellations, DD observation residuals of GEO C04, IGSO C08, MEO C14 and GPS G20 are analyzed and displayed in Figure 5 and Figure 6. As multipath is a kind of low frequency signal, the 8th Daubechies (db8) wavelet [3] is used to recover the multipath signals.

Figure 5a shows the multipath signals during three consecutive days (DOY-311–313), indicating that the multipath errors of GEO satellites vary systematically rather than being a constant offset. It also shows that the DD observation residuals of GEO satellites during three consecutive days are strongly correlated with each other (see Table 3), and the maximum correlation coefficients are over 0.92, indicating that the multipath errors of GEO have strong repeatability in two successive days.

In addition, the multipath errors of IGSO C08 for DOY 311–313(see Figure 5b) are larger than that of GEO, which also can be seen from Table 4. The standard deviations of IGSO satellites are over 6.9 mm, whereas those of GEO satellites are less than 4.5 mm. Further, the IGSO multipath variation shows great similarity over the consecutive days, which reflects a strong correlation (see Table 3).

The DD observation residuals of MEO C14 are displayed in Figure 6a. Different from the two satellites (GEO and IGSO) discussed above, the variation and RMS value of MEO multipath errors have no relationship over consecutive days, but shows great agreement in DOY 306 and 313. Table 3 also shows that the correlate time shifts calculated based on the maximum correlation are similar to the calculation in Table 1 and Table 2, indicating that the time shifts are correctly calculated.

## 5. BDS Multipath Mitigation in Dynamic Deformation Monitoring

We used two methods to mitigate BDS multipath errors. One is the sidereal filtering in the coordinate and observation domain, the other is the MHM model [20]. The coordinate domain sidereal filtering extracts multipath signals from the coordinate residuals of the first day to establish multipath model. And then the multipath model is subtracted from the raw coordinate time series in the subsequent day to get the multipath-free solutions. The observation domain sidereal filtering extracts multipath signals from the double difference carrier-phase residuals as the observation multipath models. Then the multipath models are subtracted from the corresponding double-difference phase observations of the subsequent day for GEO and IGSO satellites, and the seventh day for MEO satellites. Since GEO satellites are basically stationary and the five GEO satellites are visible all day in most areas of China, we fix the reference satellite to avoid frequent change of reference satellite in observation domain sidereal filtering. C03 satellite has a high elevation angle (about 57º) among the five GEO satellites and has little multipath signals, so we take C03 satellite as the fixed reference satellite.

**Figure 7:**
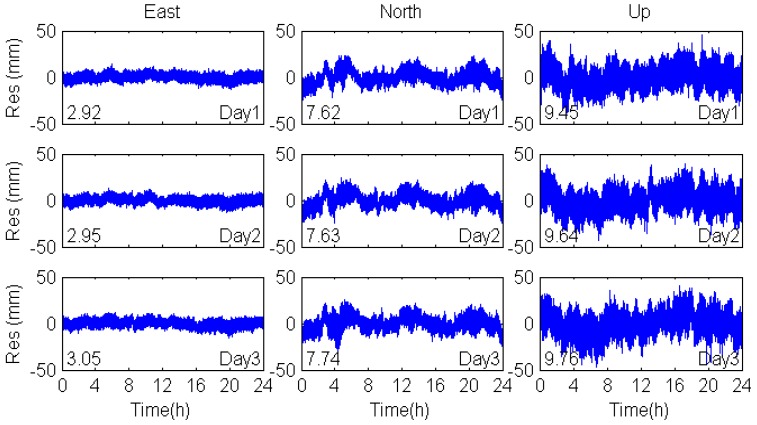
Residuals of the original coordinate sequences in the east, north and vertical coordinate directions. Numbers in the lower left corners are the RMS value of the coordinate residuals.

As Figure 7 shows, the original coordinate sequences in the east, north and vertical coordinate directions all show great similarity and agreement with those of the consecutive days, indicating the periodical change of multipath effects. In addition, we can also see that the east coordinate direction has the highest precision in three coordinate directions, and that is because five GEO satellites are located in the east-west direction.

**Figure 8:**
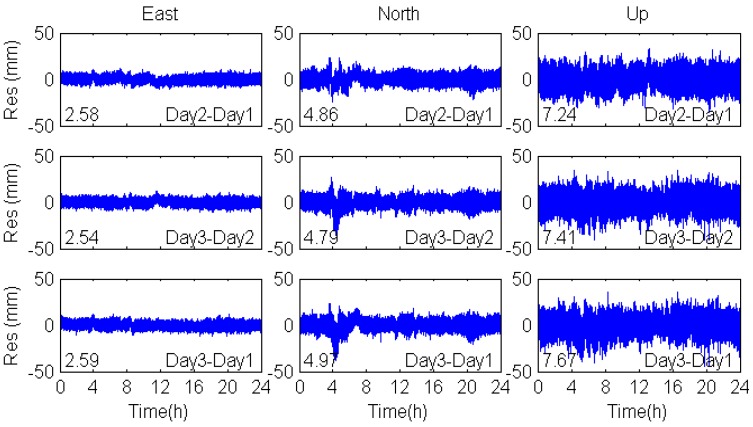
Sidereal filtering results in the coordinate domain for multipath errors in the east, north and vertical coordinate directions.

**Figure 9:**
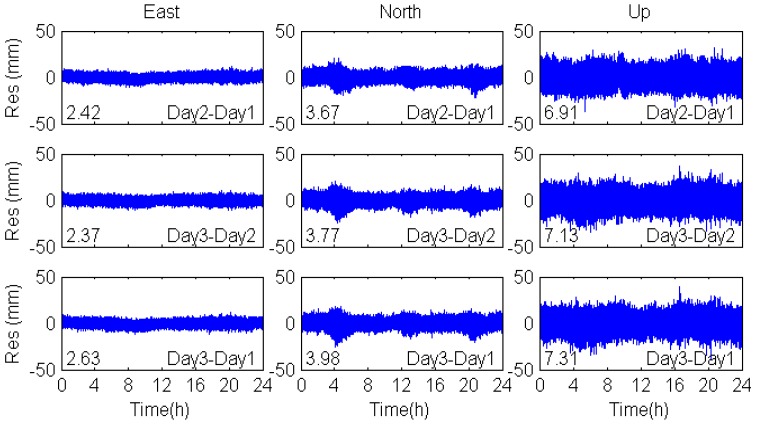
Sidereal filtering results in the observation domain for multipath errors in the east, north and vertical coordinate directions.

Figure 8 shows that the filtered residuals of coordinate time series in the east, north and vertical coordinate directions in the coordinate domain are smoother than the unfiltered one (see Figure 7). There are some peaks in the north direction, which are caused by the MEO multipath. As is discussed above, the orbital repeat periods of MEO satellites are seven days, so that the consecutive days’ multipath errors of MEO satellites are not significantly correlated with each other. Therefore, the multipath errors of MEO satellites cannot be eliminated by coordinate domain filtering. For further comparison, the observation domain filtering was also applied to mitigate multipath errors and the coordinate residuals are shown in Figure 9. The correlation among the multipath effects is shown in Table 5, including the correlation coefficients, RMS values and percentage improvements.

The correlation coefficients of DOY-312-311 and DOY-313-312 in the east, north and vertical coordinate directions are over 0.81, while the correlation coefficients of DOY-313-311are smaller. These results confirm the repeatability of multipath effects when the environment of the station remains unchanged. In addition, the RMS values of the filtered positioning coordinate time series based on observation domain are smaller than that based on coordinate domain. We can conclude that the observation domain filtering is superior to the coordinate domain filtering.

**Figure 10:**
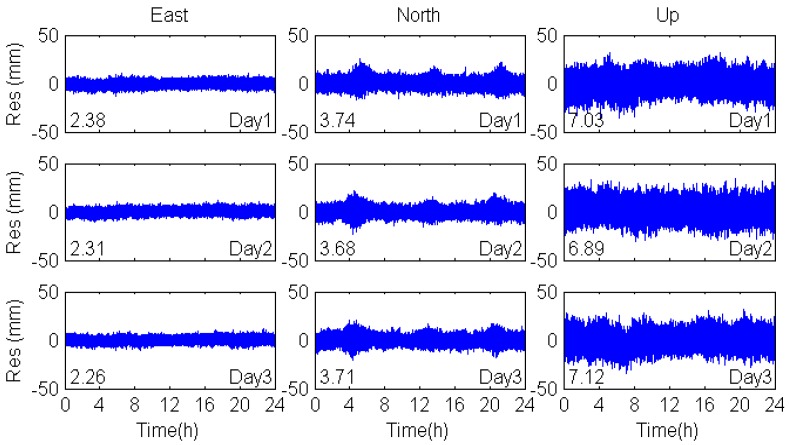
MHM model filtering results for multipath errors in the east, north and vertical coordinate directions.

The second method is based on the MHM model, which was proposed for mitigating GPS multipath effects [20]. As the GEO satellites are almost stationary and five of them are visible all day in most areas of China, we utilized the double difference model instead of the single difference model [20] to establish the BDS MHM model [31]. As the repeat periods of MEO satellites are seven sidereal days, we used observations over seven sidereal days (DOY 299-305) to establish the BDS MHM model, which was then used to mitigate multipath effects for DOY 306-313. The MHM model filtering results of three consecutive days are shown in Figure 10.

As shown in Figure 10, the filtered coordinate residuals, shown as white noise, are smoother than the unfiltered (Figure 7). As is shown in Table 6, the RMS of coordinate residuals was effectively reduced, especially in the north direction that was affected seriously by the multipath signals.

## 6. BDS Multipath Mitigation in Precise Static Positioning

The multipath errors of GPS are random for long time observations, so that they can be mitigated in high-precision static measurement. However, the GEO satellites are nearly stationary, which means they are more susceptible to multipath errors with a systematic shift. To investigate the multipath effects on BDS static baseline solutions, the GPS and BDS observations in strong and weak multipath environment were processed by a static model, respectively, and the results are compared with GPS static baseline solutions, which are showed in Table 7 and Table 8.

The difference between BDS and GPS static solutions is significant (see Table 8), while the BDS and GPS static solution results are basically consistent in weak multipath environment (see Table 7), which is less than 3 mm. As this baseline is only 6.5m, many error sources, such as orbit error, clock error of receivers and satellites, ionosphere errors, and tropospheric delay, can be mostly mitigated using double-difference technique. Therefore, the main difference is caused by the multipath errors of GEO satellites with a systematic shift.

To mitigate the multipath effects on the static baseline solutions, a new mitigation method is developed. Since the GEO satellites are nearly stationary, the multipath errors of GEO satellites change slowly with small amplitudes. So we added a parameter (Z) to the adjustment model, and the modified adjustment model is expressed as:

(6) V=Ax+By+Cz−L

where A,B,V,x,y and L are the same as those in Equation (5), z is the added systematical parameter, C is the coefficient of the added systematical parameter. We used the modified adjustment model to mitigate GEO multipath errors and the results are shown in Table 9.

The multipath effects on static baseline solutions have been effectively mitigated by the modified adjustment model, especially in the vertical direction which was contaminated seriously by the multipath. The average improvement rates are 82%, 54% and 68% in the east, north and vertical coordinate directions, respectively. Although the results in the vertical direction have been improved, the difference between BDS and GPS static solutions is still more than 4 mm. There may be two reasons: first, the correlation between the added systematical parameter and the baseline vectors was not considered in this model; second, we took the mean values of GEO multipath errors as the additional systematical parameter, but the multipath errors of GEO satellites were varied in practice.

## 7. Conclusions

We studied the characteristics of BDS orbital repeat periods and multipath errors of three different constellations (GEO, IGSO and MEO satellites), and compared the effects of sidereal filtering and MHM model in mitigating multipath for BDS dynamic deformation monitoring. In addition, the effects of BDS multipath on static solutions and its mitigation method were investigated. Some conclusions can be drawn as follows.

The multipath repeat periods of GEO and IGSO satellites are approximately one sidereal day, while it is around seven sidereal days for MEO satellites. The multipath errors of BDS are not only systematic but also random, but those of GEO are mostly systematic with a shift all the time.

BDS multipath signals can be effectively mitigated by both the MHM model and sidereal filtering. In the experiment, the improvements of coordinate domain sidereal filtering on coordinate time series are 15%, 37% and 24% in the east, north and vertical coordinate directions, respectively. The improvements of observation domain sidereal filtering and MHM model are 18%, 51%, 27% and 22%, 52%, 27%, respectively. Obviously, the MHM model is more effective.

The BDS multipath errors on static solutions are a few centimeters in multipath-rich environments, and the modified adjustment model with an additional systematical parameter can reduce the effects of GEO multipath errors on static solutions by 82%, 54% and 68% in the east, north and vertical coordinate directions, respectively.

However, the adjustment model does not consider the correlation between the baseline vectors and the GEO multipath errors. Therefore, developing a more superior method to eliminate the effects of GEO multipath errors on static baseline solutions will be our future work.

## Figures and Tables

**Table 1:** Time shift of GEO and IGSO over two consecutive days (unit: s).

PRN	Ta1(311–312)	Tg1(311–312)	Ta2(312–313)	Tg2(312–313)
C01	234	-	240	-
C02	237	-	238	-
C03	235	-	240	-
C04	237	-	236	-
C05	236	-	235	-
C06	263	252	262	249
C07	221	228–230	221	227–229
C08	239	236–240	237	236–239
C09	246	224–246	250	246
C10	231	239,240	231	239,240

**Table 2:** Time shift of MEO for DOY 305, 312 and 306, 313 (unit: s).

PRN	Ta1(305–312)	Tg1(305–312)	Ta2(306–313)	Tg2(306–313)
C11	1704	1702	1704	1704
C12	1704	1703	1704	1704,1705
C14	1704	1704	1704	1702,1703

**Table 3:** Double difference residuals for C04, C08 and C14.

DOY	C04		C08		DOY	C14	
	Correlation Coefficient	Time Shift (s)	Correlation Coefficient	Time Shift (s)		Correlation Coefficient	Time Shift (s)
311–312	0.9375	233	0.9847	231	311–312	0.0089	-
312–313	0.9477	256	0.9721	231	305–312	0.9156	1706
311–313	0.9263	463	0.9463	275	306–313	0.9076	1705

**Table 4:** Standard deviations of GEO, IGSO and MEO (unit: mm).

DOY	C04	C08	C14
311	4.1	7.1	15.2
312	3.9	7.0	11.4
313	4.3	6.9	13.7
average	4.1	7.0	13.4

**Table 5:** Correlation of the BDS multipath effects.

DOY	Coordinate	Max Correlation Coefficient	Time Shift (s)	RMS (mm)	Coordinate Domain		Observation Domain	
					RMS (mm)	Improvement (%)	RMS (mm)	Improvement (%)
312–311	E	0.8952	239	2.95	2.58	12.5	2.42	18.0
	N	0.9103	215	7.63	4.86	36.3	3.67	51.9
	U	0.8761	235	9.64	7.24	24.9	6.91	28.3
313–312	E	0.8129	248	3.05	2.54	16.7	2.37	22.3
	N	0.8922	217	7.74	4.79	38.1	3.77	51.3
	U	0.8971	236	9.76	7.41	24.1	7.13	26.9
313–311	E	0.7851	467	3.05	2.59	15.1	2.63	13.8
	N	0.8154	444	7.74	4.97	35.8	3.98	48.6
	U	0.8659	486	9.76	7.67	21.4	7.31	25.1

**Table 6:** RMS of coordinate residuals.

DOY	Coordinate	Original	MHM	Improvement (%)
		RMS (mm)	RMS (mm)	
Day1	E	2.92	2.38	18.5
	N	7.62	3.74	50.9
	U	9.45	7.03	25.6
Day2	E	2.95	2.31	21.7
	N	7.63	3.68	51.8
	U	9.64	6.89	28.5
Day3	E	3.05	2.26	25.9
	N	7.74	3.71	52.1
	U	9.76	7.12	27.0

**Table 7:** Comparison of BDS and GPS static solutions in weak multipath station (unit: mm). dE, dN and dU represent the difference between BDS and GPS static baseline solutions.

DOY	dE	dN	dU
292	1.4	1.6	2.5
293	1.6	0.6	1.9
294	1.6	1.6	2.4
295	1.6	1.4	2.5
296	1.5	0.9	2.2
297	1.8	1.4	2.8
298	1.8	1.3	2.3

**Table 8:** Comparison of BDS and GPS static solutions in multipath-rich station (unit: mm). dE, dN and dU represent the difference between BDS and GPS static baseline solutions.

DOY	dE	dN	dU	DOY	dE	dN	dU
300	7.9	4.4	16.6	307	6.6	5.0	17.0
301	5.8	6.0	16.7	308	3.8	1.2	8.7
302	4.7	5.3	16.3	309	3.3	1.4	9.7
303	5.2	5.1	16.0	310	2.6	2.0	9.7
304	6.1	5.1	16.7	311	3.1	2.0	10.7
305	7.0	5.0	16.1	312	3.0	2.4	10.5
306	7.4	4.7	15.5	313	2.9	2.0	10.1

**Table 9:** Comparison of BDS and GPS static baseline solutions before and after using the new model (unit: mm). dE1, dN1 and dU1 represent the original difference between BDS and GPS static baseline solutions. dE2, dN2 and dU2 represent the new difference between BDS and GPS static baseline solutions using the modified adjustment model.

DOY	dE1	dE2	Improvement (%)	dN1	dN2	Improvement (%)	dU1	dU2	Improvement (%)
300	7.9	1.9	75.9	4.4	1.8	59.1	16.6	6.6	60.2
301	5.8	0.4	93.1	6.0	1.3	78.3	16.7	1.9	88.6
302	4.7	0.8	83.0	5.3	0.8	84.9	16.3	0.5	96.9
303	5.2	0.1	98.1	5.1	1.7	66.7	16.0	4.9	69.4
304	6.1	0.1	98.4	5.1	1.8	64.7	16.7	6.1	63.5
305	7.0	1.4	80.0	5.0	2.4	52.0	16.1	9.4	41.6
306	7.4	2.6	64.9	4.7	2.4	48.9	15.5	8.2	47.1
307	6.6	1.3	80.3	5.0	2.4	52.0	17.0	8.0	52.9
308	3.8	1.6	57.9	1.2	1.0	16.7	8.7	4.2	51.7
309	3.3	0.8	75.8	1.4	1.3	7.1	9.7	2.6	73.2
310	2.6	0.5	80.8	2.0	1.5	25.0	9.7	1.4	85.6
311	3.1	0.1	96.8	2.0	1.6	20.0	10.7	1.1	89.7
312	3.0	0.4	86.7	2.4	2.4	0.0	10.5	2.8	73.3
313	2.9	0.3	89.7	2.0	1.8	10.0	10.1	1.8	82.2
average	5.0	0.9	82.0	3.7	1.7	54.1	13.6	4.3	68.4
